# Genetics and Epigenetics in Neoplasms with Plasmacytoid Dendritic Cells

**DOI:** 10.3390/cancers14174132

**Published:** 2022-08-26

**Authors:** Florian Renosi, Mary Callanan, Christine Lefebvre

**Affiliations:** 1 INSERM, EFS BFC, UMR1098 RIGHT, University of Bourgogne Franche-Comté, F-25000 Besancon, France; 2 Laboratoire d’Hématologie et d’Immunologie Régional, Etablissement Français du Sang Bourgogne Franche-Comté, F-25000 Besancon, France; 3 INSERM 1231 and 1209, University of Bourgogne-Franche Comté, F-21000 Dijon, France; 4 Service d’Oncologie Génétique, CHU Dijon Bourgogne, F-21000 Dijon, France; 5 INSERM 1209 and CNRS UMR 5309, Université Grenoble-Alpes, F-38000 Grenoble, France; 6 Laboratoire de Génétique des hémopathies, Institut de Biologie et de Pathologie, CHU Grenoble Alpes, F-38000 Grenoble, France

**Keywords:** blastic plasmacytoid dendritic cells neoplasm, mature plasmacytoid dendritic cells proliferation, acute myeloid leukemia, *RUNX1* mutation

## Abstract

**Simple Summary:**

Differential diagnosis between Blastic pDC Neoplasm (BPDCN) and Acute Myeloid Leukemia with pDC expansion (pDC-AML) is particularly challenging, and genomic features can help in diagnosis. This review aims at clarifying recent data on genomics features because the past five years have generated a large amount of original data regarding pDC neoplasms. The genetic landscape of BPDCN is now well-defined, with important updates concerning *MYC/MYC* rearrangements, but also epigenetic defects and novel concepts in oncogenic and immune pathways. Concerning pDC-AML, they now appear to exhibit an original mutation landscape, especially with *RUNX1* mutations, which is of interest for diagnostic criteria and for therapeutic purposes. We highlight here these two different profiles, which contribute to differential diagnosis between BPDCN and pDC-AML. This point is particularly important for the study of different therapeutic strategies between BPDCN and AML.

**Abstract:**

Plasmacytoid Dendritic Cells (pDC) are type I interferon (IFN)-producing cells that play a key role in immune responses. Two major types of neoplastic counterparts for pDC are now discriminated: Blastic pDC Neoplasm (BPDCN) and Mature pDC Proliferation (MPDCP), associated with myeloid neoplasm. Two types of MPDCP are now better described: Chronic MyeloMonocytic Leukemia with pDC expansion (pDC-CMML) and Acute Myeloid Leukemia with pDC expansion (pDC-AML). Differential diagnosis between pDC-AML and BPDCN is particularly challenging, and genomic features can help for diagnosis. Here, we systematically review the cytogenetic, molecular, and transcriptional characteristics of BPDCN and pDC-AML. BPDCN are characterized by frequent complex karyotypes with recurrent *MYB/MYC* rearrangements as well as recurrent deletions involving *ETV6*, *IKZF1*, *RB1*, and *TP53* loci. Epigenetic and splicing pathways are also particularly mutated, while original processes are dysregulated, such as NF-kB, TCF4, BCL2, and IFN pathways; neutrophil-specific receptors; and cholinergic signaling. In contrast, cytogenetic abnormalities are limited in pDC-AML and are quite similar to other AML. Interestingly, *RUNX1* is the most frequently mutated gene (70% of cases). These typical genomic features are of potential interest for diagnosis, and also from a prognostic or therapeutic perspective.

## 1. Introduction

Plasmacytoid Dendritic Cells (pDC), the natural type I interferon producing cells, are key players in innate and adaptive immune responses. Two major types of neoplastic counterparts for pDC are now discriminated by the *World Health Organization (WHO) Classification of Tumours of Heamatopoietic and Lymphoid Tissues*: Blastic pDC Neoplasm (BPDCN) and Mature pDC Proliferation (MPDCP), associated with myeloid neoplasm [1,2]. The first is a rare, aggressive, and well-documented neoplasm, initially described as CD4^+^ CD56^+^ neoplasm, with frequent skin involvement and leukemic dissemination, mainly affecting elderly men [1,3,4,5,6,7]. Conversely, MPDCP are still insufficiently described: they have only been recently introduced as a distinct entity in the 5th edition of the *WHO Classification of Tumours of Heamatopoietic and Lymphoid Tissues* [1,2]. Two types of MPDCP are now better described: Chronic MyeloMonocytic Leukemia with pDC expansion (pDC-CMML) and Acute Myeloid Leukemia with pDC expansion (pDC-AML). The aim of this review is to clarify recent data on their genomics features because the past five years have generated a large amount of original data on pDC neoplasms. The genetic landscape of BPDCN is now well-defined (*MYC/MYC* rearrangements, epigenetic defects, oncogenic and immune pathways), while pDC-AML now appear to exhibit an original mutation landscape, which is of interest for diagnostic criteria. Consequently, genetic data are now sufficiently described to allow comparison, and we highlight here the key defects, which is particularly important for diagnosis, prognosis, and future therapeutic strategies.

## 2. Genetics of Blastic Plasmacytoid Dendritic Cell Neoplasms

Cytogenetic abnormalities are detected in 57 to 75% of BPDCN patients. Most of the karyotypes contain a wide spectrum of cytogenetic abnormalities, leading to a complex karyotype (CK) (≥3 aberrations) in more than 50% of cases [8,9]. Abnormal karyotypes of BPDCN show a high number of aberrations (mean = 6.8 per case). Using conventional cytogenetic and Fluorescent In Situ Hybridization (FISH)/multi-FISH approaches, a special and distinct cytogenetic signature of BPDCN have been described, showing various but recurrent chromosomal losses or deletions over gains. These abnormalities include 6 major recurrent chromosomal losses detected at high frequency among abnormal karyotype: 5q deletion (72%), 12p deletion (64%), 13q deletion or monosomy 13 (64%), 6q deletion (50%), 15q deletion or monosomy 15 (43%), and monosomy 9 (28%) [8]. Remarkably, among CK, careful examination revealed that three or more of these six chromosomal targets were associated in 50% of cases, defining a special cytogenetic signature for BPDCN (Table 1). These results have been confirmed by two independent studies [10,11].

These recurrent deletions were confirmed by chromosomal microarrays analyses, with losses of 9p21.3 (*CDKN2A/CDKN2B*), 12p13.2-p13.1 (*CDKN1B*, *ETV6*), 13q11-q21 (*LATS2*, *RB1*), 5q31 (*NR3C1*), or 7p12.2 (*IKZF1*) [8,9,12]. Despite this original pattern of recurrent abnormalities, there is no unique key genetic event in BPDCN. Indeed, at least four genes are particularly deleted and/or mutated: *IKZF1*, required for BPDCN differentiation [13]; *RB1*, potentially associated with transformation in the case of biallelic inactivation [14], *ETV6*, whose deletion would correspond to an early pathogenic event [11], and *NR3C1*, involved in the glucocorticoid metabolism [10]. The transcriptional activators *MYC* and *MYB* are also originally rearranged at high frequency in this neoplasm. Remarkably, the significant association of these alterations (i.e., loss of *CDKN2A-B*/9p21, *CDKN1B*/12p13, or *RB1*/13q14, rearrangement of *MYC*/8q24 or *MYB*/6q23) constitute the special and unique pattern frequently detected in BPDCN (Table 1).

### 2.1. Deletions Involving Immune Genes

Interestingly, these recurrent deletions also involve genes related to the original function of pDC, i.e., immune response, especially losses of 6q23 (*IFNGR1*, *TNFAIP3)*, 9p21.3 (cluster of *IFNA* genes), and 12p13.2-p13.1 (*CLEC2B*, *CLEC4C*, *CLEC4E*, *TNFRSF1A)* [15]. Thus, those deletions compromise the normal function of the cells of origin of BPDCN.

### 2.2. Inactivation of Genes Encoding Cell-Cycle Inhibitors and Tumor Suppressor Genes

Similarly to other hematological malignancies, deletions inactivating Tumor Suppressor Genes (TSG), such as *TP53* [16,17,18,19], responsible for genetic instability, are also found in BPDCN. The tumor suppressor gene ATM, mutated in lymphoproliferative syndromes [20], may also be mutated in BPDCN [19], while *RB1*, involved in the regulation of the G1/S cell cycle transition, is also frequently deleted [12,17,19,21]. Initially described in retinoblastoma, in which it modeled the principle of TSG [22], *RB1* is also reported in chronic lymphocytic leukemia and Acute Lymphoblastic Leukemia (ALL) [23]. Additionally, the *CDKN1B/CDKN2B/CDKN2A* genes have a role in the G1/S transition, and their deletions are reported in some studies [9,12,17,19,24], similar to ALL [23] and lymphoma [25]. This alteration in cell cycle regulation could have a crucial role in the oncogenesis of BPDCN [17].

### 2.3. Recurrent Deletions in 5q31

Deletion in 5q are particularly recurrent in BPDCN, constituting a specific defect compared to other hematological malignancies [10,26]. In the 5q23.3 Common Deleted Region (CDR), *HINT1* was first proposed to be a key gene [26]. Indeed, *HINT1* encodes a homodimeric purine phosphoramidase, suggesting a transcriptional modulatory role. Moreover, *HINT1* deficiency would impair ATM function and thus DNA repair [26]. On the other hand, the 5q31 locus would also be a key region on chromosome 5, with deletions delineating a group of unfavorable prognostic impacts [10]. The glucocorticoid receptor gene *NR3C1* was found to be recurrently deleted, leading to haploinsufficiency and decreased glucocorticoid receptor activity [10]. The deletions impact the polycomb complex, in particular *EZH2*, with dysregulation of the *HOXA* locus and plasmacytoid dendritic differentiation.

### 2.4. Deletions of Transcription Factors

Similarly to other hematological neoplasms, transcription factor are particularly impacted in BPDCN. *ETV6* (*TEL*) is frequently mutated or deleted [8,12,15,16,17,24], contrasting with classical defects in other leukemia where translocations are more frequent, including t(12;21)(p13;q22) *ETV6::RUNX1* in B-cell Acute Lymphoblastic Leukemia (B-ALL) [27], t(5;12)(q32;p13) *ETV6::PDGFRB*, t(9;12)(p24;p13) *ETV6::JAK2* or t(4;12) (q12;p13) *ETV6::PDGFRA* in hypereosinophilic syndromes, and other translocations in rare cases of Acute Myeloid Leukemia (AML) [28]. ETV6 invalidations are, however, known in leukemia: somatic mutations of *ETV6* remain rare in AML [28], but germline mutations are also possible in the context of thrombocytopenia predisposing to AML, Myelodysplastic Syndromes (MDS), Chronic MyeloMonocytic Leukemia (CMML), B-ALL, or multiple myeloma [1].

The IKAROS family (*IKZF1/2/3*) is also frequently deleted [9,16,21,29], similarly to ALL [30], where it compromises lymphoid differentiation [31]. *ZEB2* may also be altered in BPDCN [32]. This transcription factor is involved in the commitment and lineage fidelity of myeloid and lymphoid cells at various stages of hematopoiesis and is thought to play a key role in the development of various types of AML, ALL, and lymphoma [33].

While translocations involving transcription factors have been widely described in ALL and AML, recurrent rearrangements in BPDCN were rare before 2017. Indeed, *KMT2A* (*MLL*) rearrangements had previously been described in rare cases of CD4+ CD56+ neoplams identified as BPDCN (*KMT2A::ENL* and *KMT2A::MLLT1*) [34,35], but these descriptions in BDPCN have been challenged because these reported cases do not fulfill the current diagnostic criteria of BPDCN. Indeed, these cases could correspond to CD4+ CD56+ AML, because they constitute a delicate differential diagnosis for BPDCN [36].

### 2.5. Recurrent MYC Rearrangements

Initially, the translocation t(6;8)(p21;q24) was sporadically reported by several studies, using conventional karyotyping. Since 2018, *MYC* rearrangements (8q24) have been largely described in approximately 30% of BPDCN [29,37,38,39], with a more frequent immunoblastoid morphology and sometimes a CD56-negative phenotype [37,40].

These *MYC* abnormalities bring BPDCN closer to high-grade B lymphoma [9,13,37,41], but the gene partners are really different, with specific partners that could point towards pDC differentiation. Indeed, among *MYC* rearranged cases of BPDCN, Sakamoto et al. confirmed the high prevalence of the t(6;8)—detected in 56% of cases involving the *RUNX2* locus at 6p21. Interestingly, Kubota et al. showed that the t(6;8) juxtaposes the promoter of *MYC* to the pDCs-specific *RUNX2* super-enhancer, leading to overexpression of *MYC*. In this recurrent t(6;8)(p21;q24), both *MYC* and *RUNX2* are dysregulated, and cooperate together to promote survival and proliferation of the BPDCN cells. Remarkably, *RUNX2* is physiologically involved in differentiation and migration of pDCs and plays a dominant role in controlling transcription networks in BPDCN [42].

Other partners of *MYC* have been sporadically reported but not clearly identified (i.e., 2p12, Xq24, 3p25, 14q32). It remains to be determined if *MYC* rearrangement could constitute a primary or secondary genetic event in BPDCN. In this way, the t(6;8)(p21;q24) cannot be considered as a specific genetic abnormality of BPDCN because it has been reported in follicular lymphoma [43]. Lastly, a unique study showed the adverse impact of *MYC* rearrangement, and this prognostic impact still needs to be confirmed by further independent studies [37].

### 2.6. Recurrent MYB Rearrangements

In 2017, other recurrent rearrangements were described in nine of fourteen patients, including five children [21]. Remarkably, all five children included in this series had a *MYB* rearrangement. Of note, the previous largest report of pediatric BPDCN cases exhibited several cases with 1q and/or 6q abnormalities, or translocation t(1;6)(q21;q23) [44]. These observations reveal a striking link between pediatric BPDCN and *MYB* rearrangement.

**Figure 1:**
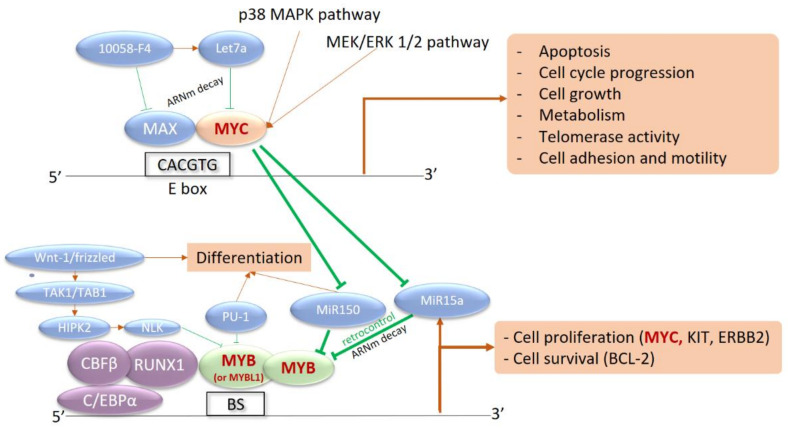
Interaction network between MYB, MYC, and their transcriptional targets. MYC is a strong transcriptional activator, dependent on the intracellular phosphorylation cascade signaling pathways of Mitogen-Activated Protein Kinases (MAPK) and Extracellular signal-Regulated Kinases (ERK) (p38 MAPK and MEK/ERK pathways 1 and 2). Activation of MYC induces the transcription of numerous target genes involved in proliferation, division, metabolism, and cell motility, as well as apoptosis. MYC also inhibits microRNAs (MiR150 and MiR15a) that are capable of silencing MYB expression in the basal state. Activation of MYC therefore induces activation of MYB, involved in the core binding factor (CBF) complex with CBFB, RUNX1, and CEBPA. MYB is also a transcriptional activator recognizing multiple nucleotide sequences, in a complex with CEBP. The targeted genes are involved in survival with BCL-2 and cell proliferation with c-KIT, c-ERBB2, and especially MYC. This results in an activation loop between the two transcriptional activators: BS, MYB Binding Site with MYB Recognition Element.

MYB rearrangements create fusion transcripts between *MYB* and various partner genes (*ZFAT*/8q24, *PLEKHO1*/1q21, *DCPS*/11q24, *miR-3134*/3p25) [21]. The chimeric transcripts retain the MYB transactivation domain and disrupt its negative regulatory domain, which allows the maintenance of the *MYB* transcriptional activity. Indeed, functional analysis of *MYB* fusions revealed the activation of *MYB* target genes as a result of induced *MYB* activation [21]. *MYB* is a nuclear-localized transcriptional activator in hematopoietic cells that interacts with the C/EBP complex to stimulate the transcriptional activity of *MYC*, *BCL2*, *c-KIT*, *c-ERBB2*, and other targets (Figure 1). Its expression progressively decreases during cell differentiation, with high activity in hematopoietic stem cells and activated T-cells.

In contrast to *MYC*, *MYB* is only exceptionally rearranged in other hematological malignancies: in fact, only in rare cases of acute basophilic leukemia with *MYB::GATA1* fusion transcripts, even rarer than BPDCN [45] and in T-ALL with t(6;7) and *MYB* duplication [46]. *MYB* translocations are also reported in 60–80% of adenoid cystic carcinomas, mainly with the *MYB::NFIB* fusion transcript [47,48], and in pediatric gliomas [49]. For the first time, fusion transcripts appear to be recurrent and specific for BPDCN compared to other hematological malignancies. *MYB* may play a key role in the leukemic transformation process, similar to *MYB::GATA1* rearrangements in acute basophilic leukemia. However, the frequency of *MYB* rearrangements is very uncertain and possibly higher in young patients, as suggested by the original study [21].

Interestingly, an analog of *MYB*, *MYBL1* would also be rearranged in a very similar way, with an identical functional impact [37]. Finally, *MYB*, *MYBL1*, and *MYC* defects would be mutually exclusive, delineating distinct groups of patients [37].

### 2.7. Mutation Landscape

#### 2.7.1. A Myeloid-like Profile

In addition to these cytogenetic defects, the mutation landscape of BPDCN has been particularly studied. However, given the rarity of BPDCN, only small cohorts have been studied so far (less than 30 cases), and usually by targeted high-throughput sequencing. Nevertheless, 22 Whole Exome Sequencing (WES) have been performed [14,16,50]. On a first stratum, the mutation landscape is quite similar to myeloid neoplasms [51,52,53,54,55,56], with a high prevalence of mutations involving epigenetics (*TET2*, *ASXL1*) and splicing (*ZRSR2*, *SRSF2*, *U2AF1*) [16,18,57,58,59]. These mutations rather suggest an early process before leukemic transformation, as is well described in AML, disturbing the DNA methylation balance, modifying chromatin access and the splicing processes [60]. In multistage leukemogenesis models, epigenetics and splicing mutations would be present from the pre-leukemic stages [61], and their frequency increases with age. Of note, mutations of *TET2* are found in 40 to 60% of cases [16,18,59]. Interestingly, loss-of-function of ZRSR2 impairs pDC activation and apoptosis after inflammatory stimuli with intron retention, promoting pDC expansion. Of note, being located on the X chromosome, this enrichment of ZRSR2 in BPDCN fits well with its predominance in males [58]. Although mutations of *NPM1* were initially described in BPDCN [16], this has not been confirmed since, and this is not consistent with the nature of these mutations defining a mutually exclusive subtype of AML [1]. In contrast, sub-clonal mutations of signaling pathways can be found in 5 to 20% of BPDCN, especially *FLT3*, *KIT*, *KRAS*, and *NRAS* mutations [16,19,21,32,50,51,59,62,63,64]. This profile is close to that of CMML and is consistent with a common clonal origin of BPDCN and CMML cells demonstrated in a few patients suffering from the two neoplasms [14,51]. The leukemic model would include shared epigenetic mutations, with secondary emergence of a BPDCN clone and another clone leading to CMML [54,65] or AML [66,67,68,69].

#### 2.7.2. Some Lymphoid-like Features

**Figure 2:**
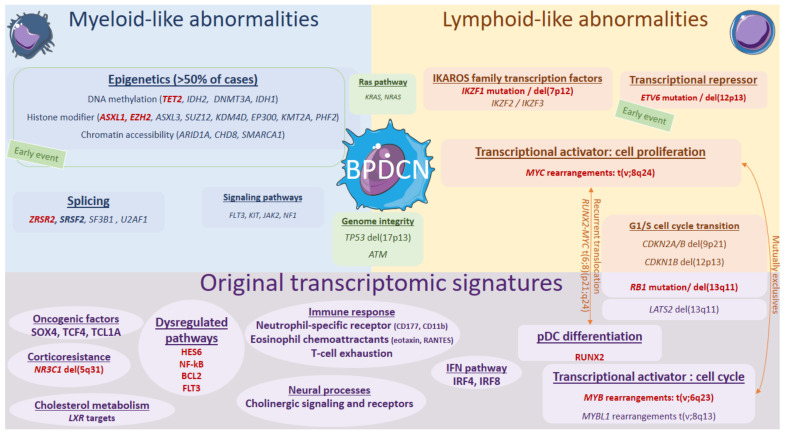
Genomics and transcriptional landscape of BPDCN. The genomic landscape of BPDCN include a combination of myeloid-like and lymphoid-like mutations and cytogenetic defects within a complex landscape, with frequent complex karyotypes. The transcriptional program of BPDCN is made of a diversity of original factors: RUNX2, MYB, IFN pathway, neural processes, cholesterol metabolism, corticoresistance factor, and original oncogenic factors.

Associated with these “myeloid-like”, key deleted transcription factors or tumor suppressor genes *IKZF1*, *ETV6*, *RB1*, *ATM,* and *TP53* can also be mutated in some cases (5–10%), also resulting in an invalidation [9,11,13,14,18,70,71]. Notably, biallelic invalidations of *ETV6* argue for a primordial early event, possibly overexpressing the BPDCN oncogene *TCL1A* [11,15,72]. *IKZF1* loss-of-function, either by deletion or mutation, would lead to the increased cell interactions in BPDCN. BPDCN also exhibit *KMT2D* and *SYNE1* mutations or losses, previously reported in follicular lymphoma [13,70]. Overall, the most characteristic feature of BPDCN would be that combination of myeloid-like and lymphoid-like abnormalities (Figure 2).

### 2.8. Prognostic Factors

A major challenge to determine the mutation landscape in BPDCN is to establish a molecular prognostic stratification, as in AML with the *European Leukemia Network* recommendation [73]. Nevertheless, this point remains a tricky issue because of the rarity and diagnostic challenges of BPDCN. Some adverse prognostic factors have still been proposed, particularly mutations involving *ETV6*, *TP53*, or *NRAS/KRAS* [16], as well as biallelic deletions of 9p21.3 [8,9], deletions of *NR3C1* [10], abnormal karyotype or numerous abnormalities detected [74,75], and rearrangements of *MYC* [37,76]. At this time, it is unfortunately impossible to integrate all of these factors into a score stratifying patients, and most of these prognostic abnormalities need to be confirmed.

## 3. Dysregulated Pathways in Blastic Plasmacytoid Dendritic Cell Neoplasms

### 3.1. Epigenetic Pathway

As the mutation profile suggests, epigenetic alterations are common in BPDCN, from DNA methylation (*TET2*, *IDH1*, *IDH2*) [16,18,19,21,32,38,50,51,54,56,57,77], to histone methylation (*ASXL1*, *EZH2*) [10,16,21,50,51,77], or chromatin remodeling (*ARID1A*, *SUZ12*) [16,21,50]. In 2019, Sapienza et al. demonstrated that the epigenetic regulatory program was the most significantly undermined in BPDCN, with shared H3K27-acetylated regions and upregulation of cell-cycle genes marked by promoter acetylation [50]. Consequently, epigenetic target strategies have been developed in BPDCN with hypomethylating agents for therapeutic purpose, with in vitro and in vitro proof of concept, and description of cases [50,78,79].

### 3.2. pDC Program with Oncogenic Deregulation

Considering their lineage on phenotypic criteria, an original transcriptional program was expected in BPDCN compared to other acute leukemia and was confirmed by various independent studies (Figure 2) [15,80,81]. For example, RB1 and LATS2 are downregulated while some oncogenes are overexpressed, such as HES6, RUNX2, and FLT3 or the NF-κB pathway [81,82]. Concordantly with the IFN pathway of pDCs, the Interferon Regulatory Factor IRF4 is overexpressed in BPDCN [81]. BPDCN are close to normal pDCs, with an enrichment in pDC and B-cell signatures, as well as in vesicular transport and deubiquitination pathways. This enrichment in B-cell signatures is also reflected by a closer proximity to B-ALL and AML on unsupervised analysis [15]. In addition, there is dysregulation of more specific transcription factors, notably SOX4, which is involved in lymphoid differentiation but also pDC by action on TCF4 [83], leading to involvement in the pDC lineage [84,85]. The anti-apoptotic factor BCL2 is also overexpressed, similarly to other hematological malignancies, opening the field of targeted therapeutic strategies [81,86,87]. Moreover, in an original way, the metabolism of cholesterol is dysregulated, namely *LXR* targets [80].

### 3.3. Emerging Concepts of Immune Response

Three different studies focused on the immune response against BPDCN and the tumor microenvironment. Indeed, Summerer et al. recently showed that a group of BPDCN up-regulated the neutrophil-specific receptors CD177 (HNA2A) and CD11b (ITGAM), indicating the presence of an activated subset of neutrophils, with a potential impact on overall survival to be confirmed [70]. On the other hand, Beird et al. showed significantly elevated levels of eosinophil chemoattractants eotaxin and RANTES in BPDCN as compared with AML, suggesting that blast cells in BPDCN are non-activated pDCs, associated with immune deficiency [88]. Finally, a recent immunologic study highlighted the T-cell exhaustion in BPDCN [89]. In a complementary approach, an infectious origin for BPDCN has been investigated, but no evidence for an induction by a virus was identified by RNA-seq on skin and bone marrow [90]. Interestingly, several dysregulated markers in BPDCN are known to be linked to neural processes [82], and a recent study especially noticed the upregulation of genes involved in the cholinergic signaling and receptors (both nicotinic and muscarinic), inducing a molecular activation of cholinergic signaling in BPDCN [91]. By analogy with solid tumors, this activation would have a negative impact on the tumor microenvironment, promoting tumor dissemination and growth [92].

To sum up, the dysregulated pathways in BPDCN are now analyzed with a microenvironment scope, and dysregulated pathways needs to be correlated to these potential immune defects.

## 4. Genetics of pDC Proliferation Associated with a Myeloid Disorder

BPDCN should not be confused with other pDC proliferation, the latter constituting a heterogeneous entity. MPDCP associated with a myeloid neoplasm corresponds to an emerging entity, referred to as a differential diagnosis from BPDCN in the 4th edition of the WHO *Classification of Tumours of Heamatopoietic and Lymphoid Tissues* [1]. Contrary to BPDCN, pDCs are morphologically mature and remarkably CD56 negative, with a low Ki-67 proliferation index (<10%), bringing them very close to normal pDCs. Recent studies show that these pDCs represent a clonal proliferation and justify them to be mentioned in the current revision (5th edition of the WHO *Classification of Tumours of Heamatopoietic and Lymphoid Tissues*) [2]. Genetically, the two better-characterized types of MPDCP are pDC-CMML and pDC-AML, where their neoplastic nature has been evidenced by shared defects between the pDCs and the myeloid neoplasm.

### 4.1. Genetics of pDC-CMML

Nodules of pDC have been commonly described in the bone marrows of CMML [93,94,95,96], but their link with the neoplasm remained uncertain until recently, when a study highlighted the clonal relationship between pDC and monocytes [97]. Of note, these nodules are very frequent in CMML (20–30% of cases) and are consequently included in all the current studies concerning CMML. Surprisingly, the most impacted pathways in pDC-CMML are not different from other CMML, with the Ras pathway (*NRAS*, *KRAS*, *NF1*, *CBL*, *PTPN11*, *PTPN13*), the epigenetic regulators (*TET2*, *ASXL1*), the splicing factors (SRSF2), and intracellular signaling pathway (*CSF3R*, *BLNK*) mutations found in both monocytes and pDCs [97]. Importantly, pDC-CMML was not found to be associated with *RUNX1* mutations, otherwise found in 30–40% of CMML [98,99]. In the current state of knowledge, there are few discriminative features of these pDC-CMML, but an accumulation of regulatory T cells and an increased risk of progression in AML should be highlighted.

### 4.2. Genetics of pDC-AML

#### 4.2.1. Cytogenetics of pDC-AML

Conversely, pDC-AML have been less described, and only in independent studies, with concordant descriptions [64,96,100,101,102,103,104,105,106]. The discrepancy between pDC-CMML and pDC-AML can be explained by the study methods used. Indeed, pDC are frequently gathered in nodules or islands, well-identified on osteomedullary biopsies, but hard to see on bone marrow smears, which is more often used in the context of AML. Moreover, the recent study of Xiao et al. estimates the incidence to be approximately 5% of AML, thus rarer than in CMML [102]. Despite these challenges, the data currently available allow us to draw a relatively comprehensive overview of the genomic landscape of pDC-AML.

At first, similarly to pDC-CMML, chromosomal abnormalities in pDC-AML seem to be similar to those found in other AML, with a clonal relationship between pDC and blast cells. This clonal relationship has occasionally been reported by karyotype or FISH: notably with isolated del(5q), monosomy 7, trisomy 8, trisomy 13, trisomy 20, or classical recurrent rearrangements such as 11q23 rearrangement involving *KMT2A* and inv(16)(p13q22) with *CBFB::MYH11* fusion [64,96,100,101,102,103,104,106,107]. In the largest cohort published to date, the landscape of cytogenetic abnormalities is similar to those described in conventional AMLs, without monosomy 9, 13, or 15, without loss of 9p, 13q, or 15q and with only 10% of complex karyotype, contrary to BPDCN [102]. Importantly, cases with inv(16)(p13q22) fall within the entity defined by the WHO *Classification of Tumours of Heamatopoietic and Lymphoid Tissues* [1] of acute myeloid leukemia with inv(16)(p13.1q22), and could not be considered as a distinct provisional entity of pDC-AML. Of note, the trisomy 20 is the only uncommon cytogenetic abnormality in AML of those descriptions, but its frequency remains uncertain, as it has only been described by one team (two cases out of eight) [101], and not found in other studies [64,102,103].

#### 4.2.2. Molecular Defects in pDC-AML

Since 2020, the only molecular abnormalities described were the presence of the *CBFB::MYH11* transcript [64,104,107] and *FLT3-Internal Tandem Duplication (FLT3-ITD)* [100,101,104,105]. Contrary to the *CBFB::MYH11* transcript defining a distinct entity, *FLT3-ITD* are sub-clonal mutations found in many subtypes of AML, and even in BPDCN [16,62]. Thus, it does not constitute a specific feature for these cases.

**Figure 3:**
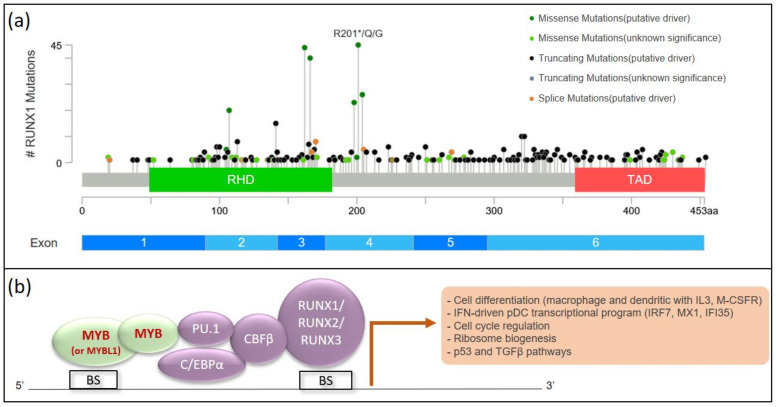
Mutation distribution and interaction network for RUNX1 and its transcriptional targets. (**a**) Mutation distribution for *RUNX1* along the gene in AML (*n* = 378 mutations, 288 patients). Adapted from www.cbioportal.org data base. RHD, runt homology domain responsible for DNA-binding and interaction with a common heterodimeric partner, CBFb; TAD, c-terminal transactivation domain. (**b**) RUNX1 interaction network: RUNX1 interacts with its binding partner CBFB, in a complex including CEBPα, PU.1, and MYB. The targeted genes are involved in cell differentiation, interferon-driven pDC transcriptional program, cell cycle regulation, ribosome biogenesis, and p53/TGFβ pathways. BS, Binding Site for RUNX family or MYB.

More importantly, two independent studies identified somatic *RUNX1* mutations in 2021 as the most common mutations in pDC-AML, detected in more than 70% of cases, whereas it was detected in 5–15% of AML and absent in BPDCN [102,103,108,109]. Thus, these cases fall within the provisional entity of AML with mutated *RUNX1* defined by the 4th edition of the WHO *Classification of Tumours of Heamatopoietic and Lymphoid Tissues* [1], with concordant clinical features and an adverse risk based on *European Leukemia Network* stratification [73]. *RUNX1* mutations have been largely studied in hematological malignancies with both germline and somatic origins. Indeed, germline mutations are reported in familial platelet disorder with predisposition to acute myeloid leukemia (FPD/AML), while somatic mutations are observed in various types of hematological malignancies, including AML. Most of the mutations were clustered in the runt homology domain (RHD) and the c-terminal transactivation domain (TAD) (Figure 3). Found in 5 to 15% of AML, *RUNX1* mutations are more frequent in elderly men, in 20–30% of secondary AML, and in 40% AML with minimal differentiation (M0-AML in the French-American-British classification) [108,110]. Interestingly, biallelic somatic mutations of *RUNX1* are strongly associated with these M0-AML [109]. Importantly, *RUNX1* mutations are an independent adverse prognostic factor in AML [110,111,112]. However, the entity of AML with somatic *RUNX1* mutation have been withdrawn from the 5th edition drops, considering its molecular heterogeneity [2]. With regard to cytogenetic abnormalities, *RUNX1* mutations are associated with trisomy 8, but also with trisomy 13 and monosomy 7/loss 7q, similarly to pDC-AML [109,111,112]. Additional mutations are found in 40 to 95% of AML with somatic mutation of *RUNX1*, especially in splicing and epigenetic factors (*SRSF2*, *SF3B1*, *ASXL1*, *IDH1/IDH2*, *TET2*, *BCOR*, *MLL-PTD*, and *DNMT3A*), with sub-clonal mutations in signaling pathways (*FLT3*, *NRAS*) [108,110,111]. In contrast, *RUNX1* mutations are almost mutually exclusive from *NPM1* and *CEBPA* mutations, concordant with their definition of distinct entities [1,110,111,112].

Of note, *RUNX1* mutations are detected in all pDC-AML with minimal differentiation (pDC-M0-AML) but not in AML with a monocytic contingent [103]. In this respect, pDC-AML with the *RUNX1* mutation would constitute a well-defined and more homogeneous entity. However, even if the two studies contain 70% of cases with *RUNX1* mutations, the American cohort contains mostly secondary monocytic pDC-AML, and a minority of undifferentiated AML, whereas the French cohort contains mainly M0-AML. Thus, two subsets of pDC-AML can be suggested, pDC-M0-AML and secondary monocytic pDC-AML [102,103]. Recently, such results were also confirmed in two cases of monocytic pDC-AML, bearing *RUNX1* and *FLT3-ITD* mutations in blast cells and in pDCs [113].

In addition to *RUNX1* mutations, classical early stage mutations in splicing genes (*SF3B1*, *SRSF2*, *U2AF1*) and epigenetic modifiers (*DNMT3A*, *TET2*) are frequent [102,103]. Moreover, pDCs, and also monocytes and conventional DCs, share the same mutations with blast cells, arguing for a common leukemic origin of all cell types [102,103].

Mechanistically, RUNX1 forms with CBFβ the core binding factor (CBF) [114], a key heterodimeric complex for hematopoiesis, interacting with CEBPA, PU.1 and MYB (Figure 3). Thus, while *MYB* may be a key gene in BPDCN, *RUNX1* appears to be involved in pDC-AML. Xiao et al. showed that leukemic blasts upregulated IFN-driven pDC transcriptional programs, particularly IRF7, MX1, and IFI35, directing toward pDC differentiation and expansion in the case of pDC-AML, but also in AML with the *RUNX1* mutation, even in the absence of pDC [102]. Moreover, among the transcriptional targets of RUNX1 are interleukin-3 and the macrophage-colony stimulating factor receptor, involved in the macrophage and dendritic differentiation, which links this with pDC differentiation [115]. Finally, invalidation of RUNX1 would lead to increased expression of RUNX2 and RUNX3 proteins, as the overall level of the RUNX family is thought to be constant and follows a compensatory mechanism in the case of a decrease in the level of one of the three proteins [116,117]. In the case of a switch from RUNX1 to RUNX2, it would promote commitment to the pDC lineage because RUNX2 is a key player in differentiation into pDCs [42,118]. Expression levels of the RUNX family would represent an interesting pathway to study in AML, as recently shown with RUNX3, found downregulated in AML with *RUNX1::RUNX1T1* transcript and upregulated in AML with normal karyotype [119]. Of note, high levels of RUNX3 would be associated with poor outcome [119,120].

## 5. Conclusions

pDC-AML constitutes a challenging differential diagnosis for BPDCN, and genomic feature can help in diagnosis (Table 1). Indeed, each neoplasm is characterized by a blast cell contingent, occurring predominantly in males after 60 years, with possible skin lesions [102,103]. In pDC-AML, a continuous maturation between blast cells and pDC has been well-documented for several years [100,101,103]. The two contingents are therefore hard to split by flow cytometry, and the detection of this continuous maturation from blasts to pDCs is a key element needed in order not to misunderstand it is a pDC-AML. Thus, some studies can still consider cases evoking pDC-AML as an immature subgroup of BPDCN, due to lack of clear diagnostic criteria, and additional criteria are required to ensure the diagnosis [100,101,103,121,122]. Remarkably, *RUNX1* is the most frequently mutated gene in pDC-AML (70% of cases) [102,103], while *RUNX1* mutations are extremely rare in BPDCN [16]. There are still few data, but it may constitute an argument in cases overlapping with BPDCN. It should be noted that BPDCN may also be associated with myeloid neoplasms, either concomitantly or sequentially [123,124]. The associated neoplasm is usually CMML [125,126,127], but it can also be a MDS [128,129,130,131]. In cases with BPDCN and CMML, a common clonal origin has been demonstrated [14,51]. Considering recent descriptions of pDC-CMML and pDC-AML, such cases are obviously of interest and also constitute a risk of misdiagnosis between the two entities. In conclusion, the typical genomic features of BPDCN and pDC-AML now appear clearly distinct, with potential interest in diagnosis (*MYC*, *MYB*, *ETV6*, *RB1* in BPDCN, *RUNX1* in pDC-AML) but above all from a prognostic or therapeutic perspective. In the era of targeted therapies, recent studies provide a rationale for the use of some of them. In BPDCN, various targeted therapies show promising results: hypomethylating agents have been suggested for epigenetic defects, Bromodomain and Extra-Terminal motif (*BET*) protein inhibitors for TCF4 expression and *MYC* rearrangements, bortezomib for activation of the NF-kB pathway, and venetoclax for the hyperexpression of BCL2 [50,78,79,84,86,132,133]. Concerning pDC-AML, BET protein inhibitors have also been proposed in AML, this time to target RUNX1 [134].

**Table 1:** Nature and frequency of cytogenetic and molecular aberrations in BPDCN compared to pDC-AML. (a) Simple karyotype: <3 cytogenetic abnormalities; (b) Complex karyotype: ≥3 cytogenetic abnormalities; mean number of cytogenetic abnormalities in BPDCN: 6.8; main defects are depicted in red; cytogenetic abnormalities are in bold while minimal deleted regions and postulated target genes are not.

Genetic Defects			BPDCN	pDC-AML
**Cytogenetic** with minimal deleted region (and postulated target genes)	**Normal karyotype**		**25 to 43%**	**38%**
	**Abnormal karyotype**		**57 to 75%**	**62%**
		**Simple karyotype (a)**	**9%**	**52%**
		**Complex karyotype (b)**	**55 to 57%**	**10%**
		**5q deletion** Including 5q31-q35 deletion (*HINT1, NR3C1*)	**30 to 72% (non-isolated)** 21 to 48%	**5% (isolated)**
		**Monosomy 7** Including 7p12 deletion (IKZF1) Including 7q deletion	19%	12% 7%
		**Trisomy 8**		5 to 25%
		**Monosomy 9** Including 9p21 deletion (*CDKN2A/CDKN2B*)	**23 to 28%** 50 to 66%	
		**12p13 deletion (*CDKN1B,* ETV6)**	**64 to 67%**	
		**Monosomy 13** Including 13q11-q14 deletion (*LATS2, RB1*)	**42 to 57%** 43 to 78%	
		**Trisomy 13**		**7%**
		**Monosomy 15**	**23 to 36%**	
		**17p13 (TP53)**	**33%**	
		**Trisomy 20**		**3 to 25%**
	References		[8,9,10,11,12,17,18]	[64,96,100,101,102,103,106,107]
**Oncogenic rearrangements**	Partner genes		***MYC::XXX*** 20–30% ***MYB::XXX*** 5–20% ***MYBL1::XXX*** 1%	***CBFB::MYH11*** < 5% **KMT2A::XXX **< 5%
	References		[21,37,39,76]	[64,104,107]
**Somatic mutations**			*TET2, ASXL1* *KMT2D, SYNE1* ***ZRSF2*** , *SRSF2, U2AF1* *FLT3, KRAS, NRAS, KIT*	*TET2, ASXL1*, *DNMT3A, IDH1, BCOR* *SRSF2, U2AF1, SF3B1* *FLT3, KRAS, NRAS, KIT*
				*CBL, NF1, PHF6, WT1*
			***IKZF1, ETV6, RB1*** *,* ***TP53**, ATM*	***RUNX1 (70%)*** *TP53*
	References		[13,16,18,19,21,32,50,51,56,57,58,59,62]	[100,101,102,103,104,105,113]
